# Epidemiology of Dental Caries and Disease Prevention Among 12-Year-Olds in Slovenia Over Thirty Years (1987–2017)

**DOI:** 10.3290/j.ohpd.a44309

**Published:** 2020-02-12

**Authors:** Vito Vrbič, Martina Vrbič, Poul Erik Petersen

**Affiliations:** a Professor, Division of Dental Medicine, Medical Faculty, University of Ljubljana, Slovenia. Study concept, idea, data acquisition, contributed substantially to the discussion, wrote the manuscript.; b ^Master of Science,^ Analyst, Private Sector, Ljubljana, Slovenia. Statistical analysis, data interpretation, study design, wrote the manuscript.; c Professor, World Health Organization Collaborating Centre for Community Oral Health Programmes and Research, Copenhagen, Denmark. Data interpretation, wrote the manuscript.

**Keywords:** children, dental caries, disease prevention, epidemiology, fissure sealing

## Abstract

**Purpose:**

The first large-scale epidemiological survey on dental caries in Slovenia was conducted in 1987 and repeated in 1993, 1998, 2003, 2008, 2013 and 2017, using the same methodology. The aim of the study was to examine the trend of caries in 12-year-olds in Slovenia during a 30-year time period. The changes over time in caries experience were compared with disease trends observed in other European countries.

**Study Populations and Methods:**

The WHO National Oral Health Pathfinder Survey was applied in all seven surveys (1987–2017). The surveys were carried out in all nine geographical regions of Slovenia. For each subject, the caries experience and presence of sealants were recorded.

**Results:**

The mean DMFT of 12-year-olds decreased significantly from 5.1 in 1987 to 1.5 in 2017 (p < 0.0001). The percentage of persons with sealed teeth increased from 6% in 1987 to 94% in 2017, and the percentage of caries-free persons increased from 6% to 42%.

**Conclusion:**

The implementation of a nation-wide preventive programme was determined to significantly contribute to the effective control of caries and continuously improve the oral health of Slovenian children. In an international perspective, the Slovenian achievements in disease prevention in terms of caries prevalence reduction may be important for other countries of the region.

Supplementary Material Reports about Dental Caries Data for Former Yugoslav Republics and Provinces Markovic N, Muratbegovic AA, Kobaslija S, Bajric E, Dragas MS, Huseinbegovic A. Caries prevalence of children and adolescents in Bosnia and Herzegovina. Acta Medica Academica 2013;42:108–116. Pezo H, Hrvatin S. Epidemiological research on oral health in Croatia, Croatian Dental Chamber [in Croatian]. 2015:1–10. Begzati A, Meqa K, Siegenthaler D, Berisha M, Mautsch W. Dental Health Evaluation of Children in Kosovo. Eur J Dent 2011;5:32–39. Manu B, Ambarkova V, Stevanovic M, Jankulovska M, Shah AF, Ishrat A et al. Dental caries experience among 12-year-old school children from Macedonia and India. J Dent Probl Solut 2015;2:44–47. Djuričković M, Ivanović M. The State of oral health in children at the age of 12 in Montenegro [in Serbian]. Vojnosanit Pregl 2011;68:1–6. Ivanović M, Carević M. Program of dental health care of the population of Serbia [in Serbian]. Proceedings of the XXVIII, XXIX and XXX Symposium of Dental Health Education. Belgrade: School of Dental Medicine, University of Belgrade, 2017:121–131.

Over the past decades, numerous epidemiological studies have been carried out which demonstrate a caries reduction in schoolchildren worldwide.^17,19^ Dental caries is one of the most common chronic diseases in children and adolescents; assessing the prevalence of caries is crucial for planning and implementation of effective preventive programmes within national healthcare systems. In addition, the social, cultural, and economic conditions that affect children’s health needs and provision of healthcare should be considered. During the past 50 years, the World Health Organization (WHO) has encouraged the use of a standard methodology for epidemiological surveys in oral health.^20^ National epidemiological surveys conducted today have made it possible to gather systematic data over time which show substantial progress in this field.

Slovenia was part of Yugoslavia until 1991. In the late 1970s, the WHO announced that only two European countries – Albania and Yugoslavia – still had no data available on the prevalence of caries. In 1985, the first author and his Yugoslav colleagues started preparation of a pilot study in cooperation with the WHO, in order to investigate the state of oral health among schoolchildren in former Yugoslavia. That study took place in 1986 in all six Yugoslav republics and the country’s two provinces. This was not only the first time, but also the only time, that data on the prevalence of caries in schoolchildren were gathered systematically in Yugoslavia, which however disintegrated five years later. The results of this first study were very discouraging. A high prevalence of caries – DMFT > 6 – was demonstrated among 12-year-olds in Slovenia and elsewhere in Yugoslavia.^26^ At the satellite symposium ‘Caries Status in Europe and Predictions of Future Trends in 1990’, it was concluded that Yugoslavia ranked at the bottom of the scale, second to last among 31 European countries.^10^

After the initial survey was carried out in 1986 in Yugoslavia, the first author of this study and his Slovenian colleagues started preparation of the first large-scale epidemiological survey on oral health in Slovenia, which took place in 1987. Our aim at that time was to evaluate the oral health status of the Slovenian population in order to determine guidelines for improving and upgrading the oral healthcare system existing at that time. In order to undertake the survey, we received a grant from the Slovenian Ministry of Education and Science. The study was performed in all nine geographical regions of Slovenia and included subjects from different age groups: 6, 12, 15, 18, 35–44, and 65 or older. For each subject, caries experience, application of pit and fissure sealants, periodontal status, and dentofacial anomalies were evaluated. The second survey was carried out in 1993 with the same scope and using the same methodology, examining subjects in the same age groups and evaluating data according to the same framework by the same examiners. Regardless of the fact that a grant was provided only for the first two surveys (1987 and 1993), the first author of the study continued to conduct five further surveys that were carried out in 1998, 2003, 2008, 2013, and 2017. However, due to lack of funding, these five surveys were limited to 12-year-olds; information about caries experience and sealant data of each subject was recorded.

The development of caries prevention began in 1956, when Slovenia (then a Yugoslav republic) was among the first in Europe to start using and producing calcium fluoride tablets.^8^ The Public Youth Dental Health Service (PYDHS) had failed to reduce caries prevalence in schoolchildren mostly due to the very low number of dentists in Slovenia at that time. Fluoride tablet distribution was entrusted to paediatric clinics and to teachers in schools. Fluoride prevention in 7- to 14-year-old schoolchildren was further enhanced in the late 1960s with a 4% NaF topical fluoride application (TFA), which dentists applied to schoolchildren’s teeth after dental restoration.^3,24^ At the proposal of the first author of this study, the Slovenian Republic Center for Public Health passed an act that all PYDHS offices were to provide children with complete restoration of carious lesions. TFA was to be used according to uniform guidelines and implemented throughout the country.^22^ Within only 3 years of implementation of this act, about 25,000 of 7- to 14-year-old children in Slovenia had received restoration of and topical fluoride prophylaxis for their teeth. This represented the first action plan for the complete restoration of carious lesions in combination with TFA in schoolchildren in Slovenia. Later, dental nurses in special school washrooms introduced usage of fluoridated dental gel where instruction in oral hygiene took place and still does today (e.g. supervised toothbrushing). After 1980, the number of dentists in the PYDHS increased and reached 30% of all dentists in Slovenia. In addition, the first specialists in paediatric dentistry completed their specialisation in 1976 and thereafter assumed leading positions in dental prevention in Slovenia.^29^

With the increased number of dentists in the PYDHS and the help from paediatric dentists, we started surveillance of caries in schoolchildren in Slovenia at a more systematic level. In the early 1980s, our preventive programme was expanded; in addition to preventive measures for caries, we also included prevention measures for periodontal disease and dentofacial anomalies. The preventive measures introduced up to that point were not enough to achieve a significant decrease in the DMFT in schoolchildren in Slovenia. Because most caries in schoolchildren occurs in pits and fissures of molars and premolars, we performed a 3-year study on the retention of fissure sealants and caries reduction in deciduous and permanent molars; this was carried out in six PYDHS offices in different regions.^25^ By recognising fissure sealing as a promising preventive measure – provided that application is carried out in time (3 months after tooth eruption) – we began preventive fissure sealing in children in Slovenia in 1980. Large-scale fissure sealing began a few years later; initially only first molars were sealed, but soon after second molars were included. Later in the 1990s, premolars were also included in large-scale fissure sealing in schoolchildren. Similar to other preventive measures that became part of Slovenia’s preventive programme, the Ministry of Health first agreed to implement fissure sealing nationwide. The National Institute of Public Health also participated in implementing large-scale fissure sealing by organising coordinators – that is, paediatric dentists to oversee the implementation of this preventive measure – in all health regions so that it would be implemented at the right time for all schoolchildren throughout the country. This procedure was carried out in all PYDHS offices and school dental clinics in Slovenia.

After 1991, when Slovenia became independent, the (oral) healthcare system underwent a transition. Political and economic changes prompted privatisation and decentralisation. Fortunately, the caries-preventive programme continued to function, and parallel epidemiological surveys were regularly conducted at 5-year intervals to examine possible changes in oral health in children in Slovenia. Therefore, the aim of this report is to describe trends in caries experience in 12-year-old children in Slovenia over a period of 30 years. It is noteworthy that this series of surveys comprises the only long-term systematically gathered data on caries prevalence in 12-year-olds in Slovenia, based on evaluation of individuals from across the country.

## Study Populations and Methods

The WHO Pathfinder method was applied for each survey of the study.^32^ Data collection was based on samples of 12-year-olds in all seven surveys and included individuals from all nine of Slovenia’s geographical and health regions. In 2017, Slovenia had a population of 2,065,895. The national survey population from Slovenian cities and towns included in all surveys in 2017 were as follows: the two largest Slovenian cities, Ljubljana (population 288,919) and Maribor (111,079), Celje (49,380), Novo Mesto (36,433), Nova Gorica (31,825), Murska Sobota (18,870), Radovljica (18,822), Piran (17,782), Ravne na Koroškem (11,302) and Metlika (8325).^23^ Individuals from primary schools in these cities and towns were included in samples of the surveys performed in order to ensure coverage of 12-year-old children from urban and rural areas. One randomly selected primary school in each of these cities and towns participated; in classes containing 12-year-old children, every third pupil alphabetically was enrolled. Examinations of the children were performed by dentists in the dental clinics located at the schools and were completed within 3 weeks. The parents of the children participating in surveys were requested to give informed consent for their child’s participation. In the last five out of seven surveys, a team of five dentists, including the first author of this study (who personally examined the children in six participating cities/towns,) examined the children clinically according to the methodology and WHO criteria^32^ using artificial light, a plane mirror, and a sharp explorer. The examinations focused on the presence of caries and fissure sealants. Caries was diagnosed at the cavitation level; the caries experience of each subject was recorded by counting the components DT, MT, and FT of the DMFT index (Decayed, Missing, and Filled Teeth). Fluoride levels in the drinking water were low: from 0.01 to 0.05 mg/l in eight towns and 0.24 mg/l in the other two. Most of the toothpastes used by the Slovenian population contained fluoride (1400–1500 ppm F).

The examiners were calibrated prior to the surveys in order to obtain consistency indices for recording caries. At the start of the study in 1987, three teams of dentists, each consisting of a paedodontist and a periodontologist, examined the same 20 subjects; for each subject, the presence of caries, application of fissure sealants, periodontal status, and dentofacial anomalies were evaluated. Because the first two surveys were extensive, containing six age groups of subjects, the interexaminer agreement was expressed on an interval scale for each category of examination. Agreement on caries experience varied between 82% and 88%. The calibration results of the first and second survey have already been compared and published,^27^ while calibration results of the last five surveys are presented using the ICC (intra-class correlation coefficient) to assess the degree to which the team of five dentists provided consistency in their recordings of the DMFT across the same 20 subjects. The resulting ICCs for the last five calibrations were all in the ‘excellent’ range: 1998 (ICC = 0.96; CI 0.93–0.98), 2003 (ICC = 0.97; CI 0.95–0.99), 2008 (ICC = 0.97; CI 0.95–0.99), 2013 (ICC = 0.98; CI 0.97–0.99), 2017 (ICC = 0.99; CI 0.97–0.99).

When comparing sample sizes for each survey (Table 1), it is important to highlight the phenomenon of decline in the number of all 12-year-olds living in Slovenia through the years of study. This is naturally due to a decline in the birth rate in Slovenia over the 30-year period. Our study included children born between 1975 and 2005. During that time, the number of births in Slovenia tended to decrease, with the exception of eight non-consecutive years, when the number of births with respect to the previous year did not decrease.^23^ In our first survey in 1987, the sample included 404 participating 12-year-olds, which represented 1.32% of the population of 12-year-old children living in Slovenia at that time (30,557 individuals). However, in the last survey in 2017, the number of 12-year-olds living in Slovenia was significantly smaller than in 1987, with only 18,733 individuals.^23^ The number of participating 12-year-olds included in the sample in 2017 was 300 individuals, which can be considered a substantially reduced sample size of participants in comparison to the first survey. Meanwhile, it is important to bear in mind that in relative terms, this number represents 1.60% of the 12-year-old population living in Slovenia in 2017, which is actually a higher percentage than in 1987, when the sample size was larger. In all seven surveys, 2202 12-year-olds were examined, of whom 1108 were girls and 1094 boys (Table 1).

**Table 1:** Sample distribution of 12-year-olds examined by sex

Year	Girls		Boys		Total	
	n	%	n	%	n	%
1987	203	50.2	201	49.8	404	100
1993	198	49.4	203	50.6	401	100
1998	131	51.0	126	49.0	257	100
2003	138	51.9	128	48.1	266	100
2008	137	50.0	137	50.0	274	100
2013	151	50.3	149	49.7	300	100
2017	150	50.0	150	50.0	300	100

The data analysis consisted of descriptive statistics, such as mean number of the DMFT and mean number of sealed teeth as well as the mean number of individual components of the DMFT: DT (decayed teeth), MT (missing teeth), and FT (filled teeth). The percentage of caries-free children (DMFT = 0) and the percentage of children with sealed teeth were calculated. The Significant Caries Index (SiC index) was determined by sorting individuals according to their DMFT values and calculating the mean DMFT value for the one-third of the examined population with the highest caries score.^2^ The Care index was determined by the equation (FT/DMFT) x 100 among individuals with DMFT > 0.^12^ Because both of the main dependent variables discussed here are not normally distributed, nonparametric tests were used for statistical analysis. The Kruskal-Wallis test was used to assess differences in the DMFT (DMFT index, each individual component DT, MT and FT, and SiC index) and in sealed teeth in all surveys. The Mann-Whitney test was used to assess differences in these variables between certain surveys and differences according to a specific characteristic of the subject, such as sex. All statistical tests were performed at the 5% significance level. For statistical analysis and preparation of Figures and Tables, Microsoft Excel 2010 and IBM SPSS Statistics 22 were used.^6^

## Results

The reduction over time in the total caries index (DMFT) of 12-year-old children demonstrates a convincing positive effect of the national dental disease prevention programme in Slovenia: the overall level of caries experience of children declined substantially by 70% throughout the surveys, from an average of 5.10 DMFT in 1987 to 1.53 DMFT in 2017 (p < 0.0001) (Table 2). As shown in Table 2, the caries experience of children only increased slightly from 2008 to 2013, but this was not statistically significant (p > 0.05). During the study period, the difference in mean DMFT was statistically significant between the first and second survey (p < 0.0001), the second and third survey (p < 0.001), and the sixth and seventh survey (p < 0.05) (Table 2).

**Table 2:** Mean DMFT, reduction of caries prevalence (%), and DMFT components (%) of 12-year-olds examined

Year	Mean DMFT	Standard deviation	95% confidence interval	Reduction in relation to 1987 (%)	Reduction in consecutive surveys (%)	Decayed component (%)	Missing component (%)	Filled component (%)
1987	5.10	3.37	(4.77 – 5.43)	–	–	24.3	3.1	72.5
1993	2.60	2.97	(2.31 – 2.89)	49.0	49.0	31.9	2.3	65.8
1998	1.79	2.14	(1.53 – 2.06)	64.9	31.2	14.5	2.2	83.2
2003	1.69	1.98	(1.45 – 1.93)	66.9	5.6	29.6	1.8	68.6
2008	1.66	1.93	(1.43 – 1.89)	67.5	1.8	37.3	1.2	61.4
2013	1.89	2.01	(1.66 – 2.12)	62.9	-13.9	22.8	2.1	75.1
2017	1.53	1.81	(1.32 – 1.74)	70.0	19.0	38.6	0.7	60.8

In all surveys but the sixth in 2013, the mean DMFT was higher among girls than boys. However, the difference by sex was significant only in the first (p < 0.01) and the fifth survey (p < 0.05). When comparing all 2202 12-year-old boys and girls examined, girls had a higher mean DMFT than boys (p < 0.005).

**Fig 1:**
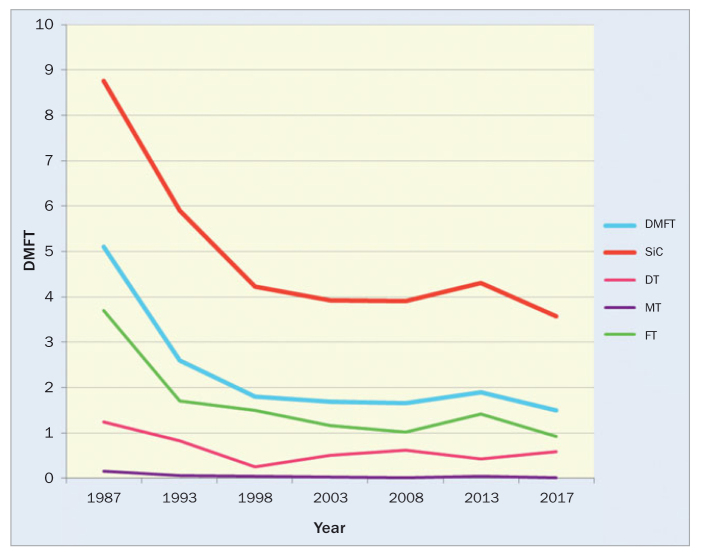
Means of DMFT, DT, MT, FT, and the SiC index (1987–2017).

The three lowest curves in Fig 1 indicate changes over time in the DMFT components: DT, MT, and FT. The DT component refers to untreated caries, and throughout the surveys it displayed different mean values (p < 0.0001), with its lowest mean being 0.26 in the third survey in 1998. Since then, somewhat higher mean values of DT were recorded, reaching a value of 0.59 in the last survey in 2017, which indicates a significant increase from the lowest point in 1998 (p < 0.0001). In the 2017 survey, an increase in the DT component was observed; the component reached the highest proportion in all surveys (38.6%), which, to say the least, was unexpected. Nonetheless, the DT component marks a considerable decline of 52.4% from the first survey in 1987 (p < 0.0001). The second component, MT, indicates the number of missing teeth that were lost due to caries. As expected, it contributed the least to the DMFT values and remained at very low levels throughout all surveys (p < 0.0001), reaching the lowest point of 0.01 in the last survey in 2017. In the last survey, the MT component also contributed the smallest proportion to the mean DMFT value of all surveys, amounting to only 0.7%, whereas the highest proportion in the mean DMFT was 0.16 in the first survey in 1987. From the first survey, the MT component declined significantly (p < 0.0001) by 93.8%. The third component (FT), refers to restorative treatment and it contributed the most to the DMFT value in all surveys (p < 0.0001). In the last survey, it reached its lowest mean value of 0.93, which shows a significant decline from the recorded value of 1.42 in 2013 (p < 0.001). The difference in mean FT is also significant when comparing the boys and girls examined in all surveys, since girls had a higher mean FT than did boys (p < 0.01). The FT component showed the highest proportion (83.2%) in the mean DMFT in the third survey in 1998.

As seen in Table 3, the SiC index continually decreased throughout the surveys (p < 0.0001) with the exception of the sixth survey in 2013, when it increased somewhat. As opposed to the insignificant increase in the mean DMFT in that same year, the SiC index increased significantly (p < 0.05) in the sixth survey in 2013 in comparison to the previous survey in 2008. However, its decrease in 2017 was also significant (p < 0.0001) in comparison to 2013. In all surveys, except the sixth in 2013, girls had a higher SiC index than boys, and the difference was significant in 1987 (p < 0.05), 1998 (p < 0.0001) and 2008 (p < 0.01). In conclusion, the SiC index in 12-year-olds in Slovenia decreased over the past 30 years (p < 0.0001), achieving a substantial decline of almost 60%.

**Table 3:** Percentage of caries-free 12-year-olds (1987–2017) and SiC index, Care Index (%)

Parameters	Year of survey						
	1987	1993	1998	2003	2008	2013	2017
SiC index	8.76	5.9	4.22	3.92	3.90	4.31	3.58
SD (SiC)	2.81	2.78	1.91	1.78	1.66	1.38	1.58
95% CI (SiC)	(8.28 – 9.23)	(5.42 – 6.37)	(3.81 – 4.63)	(3.55 – 4.30)	(3.56 – 4.25)	(4.04 – 4.58)	(3.27 – 3.89)
Care index (%)	72.6	65.6	83.1	68.7	61.4	75.1	60.8
Caries-free (%)	6.4	30.9	40.1	39.4	36.9	36.0	42.0

Table 4 presents data on the average number of sealed teeth in 12-year-olds over the period of the seven surveys. The mean number of sealed teeth increased tremendously over this time from one survey to another, with the exception of the fifth survey, as it decreased significantly in 2008 (p < 0.001). The increase was particularly steep in the first half of our study, reaching a mean number of 6 teeth; this trend reflected the incremental implementation of the programme for sealing of permanent teeth. No significant differences by sex were found in the mean number of sealed teeth in six of the seven surveys. However, the difference was significant (p < 0.05) in the fifth survey in 2008, in which girls had a higher mean number of sealed teeth (5.43) than boys (4.61). The mean number of sealed teeth among 12-year-old children in Slovenia increased markedly from the first survey in 1987 (p < 0.0001) to 2003.

**Table 4:** Mean number of sealed teeth and their increase (%), 1987–2017

Year	Mean number of sealed teeth	Standard deviation	95% confidence interval	Increase in relation to 1987 (%)	Increase in consecutive surveys (%)
1987	0.30	1.29	(0.18 – 0.43)	–	–
1993	2.76	3.88	(2.38 – 3.14)	820.0	820.0
1998	4.43	3.67	(3.98 – 4.88)	1,376.7	60.5
2003	6.07	4.04	(5.58 – 6.56)	1,923.3	37.0
2008	5.02	3.65	(4.59 – 5.46)	1,573.3	-17.3
2013	7.00	4.43	(6.50 – 7.50)	2,233.3	39.4
2017	7.11	4.51	(6.60 – 7.63)	2,270.0	1.6

When the 12-year-olds examined in each survey are divided into those that had at least one sealed tooth and those without sealed teeth, we are able to compare the value of the mean DMFT between these two groups. Even though this means comparing two very differently sized samples in each survey, the mean DMFT of the group containing individuals without sealed teeth was higher in all seven surveys. The higher mean DMFT among 12-year-olds without sealed teeth was significant in 1987 (p < 0.0001), 1993 (p < 0.0001), 2003 (p < 0.0001), and in the last survey in 2017 (p < 0.0001). A significant difference (p < 0.0001) was also found when comparing the mean DMFT in 12-year-olds examined in all surveys according to whether they had no sealed teeth (DMFT = 4.27) vs those with at least one sealed tooth (DMFT = 1.59).

The aim of the Slovenia national dental health programme has been to encourage the adoption of healthy lifestyles by children and parents through public health promotion initiatives. This includes the development of regular oral hygiene habits with use of fluoride toothpaste, improvement of parents’ concern for the oral health of their children and increasing the awareness of the importance of sugar consumption. The ultimate impact of such population-directed work would manifest in caries-free teeth. In 1987, only 6.4% of 12-year-olds were caries free, and this figure grew systematically to 42% in 2017. Thus, over the years, along with the valuable result of disease prevention, the national dental health programme has provided a significant health-promotion outcome.

At the beginning of the study the percentage of caries-free individuals did not differ much between boys (7.5%) and girls (6%). In the last survey, almost 47% of 12-year-old boys were found to be caries-free, compared to 37% of girls. A comparison between the caries-free 12-year-olds and those with DMFT > 0 shows that the mean number of sealed teeth among caries-free children was higher than among those who were not caries-free in six out of the seven surveys. The difference was significant (p < 0.0001) in the first survey, when a 12-year-old with DMFT > 0 had on average only 0.24 teeth sealed, as opposed to a caries-free 12-year-old, who had 1.2 teeth sealed. A significant difference was also found in the second (p < 0.0001), third (p < 0.001), and fourth (p < 0.0001) surveys. In the last survey, the difference in the mean number of sealed teeth between these two groups was not only significant (p < 0.0001) but also the largest, and it resulted in caries-free 12-year-olds having on average almost 9 teeth sealed. In contrast, those with DMFT > 0 had an average of 5.8 sealed teeth (Fig 3).

**Fig 2:**
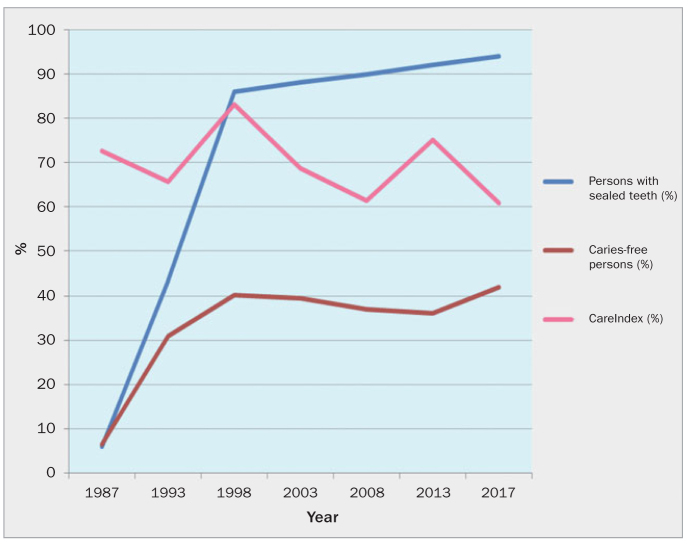
Percentage of persons with sealed teeth and caries-free persons, Care index (1987–2017).

The Care index values ranged from 60.8% to 83.1%. Regrettably, the lowest Care index value in all the surveys (60.8%) was recorded in 2017, indicating a considerable reduction in restorative care of carious teeth compared to the previous recorded Care index of 75.1% in 2013. Even in 1987, when the mean caries experience was over 5 DMFT, the Care index was as high as 72.6%. The highest value of 83.1% was found in the third survey in 1998 (Fig 2).

**Fig 3:**
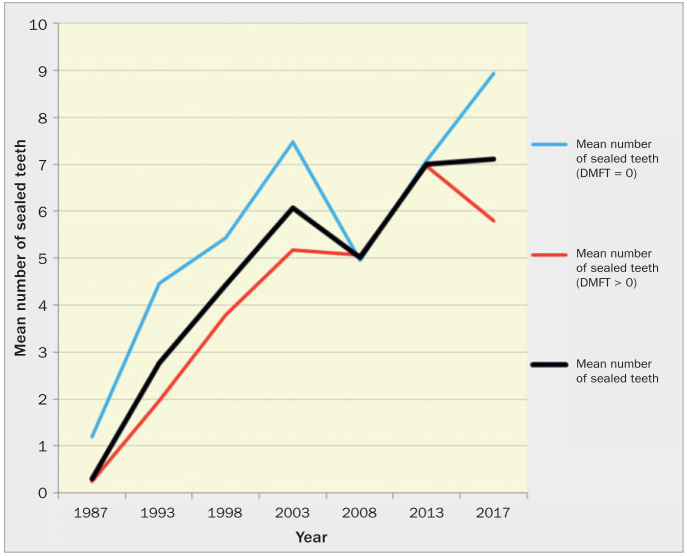
Mean number of sealed teeth for caries-free 12-year-olds, 12-year-olds with DMFT > 0, and all 12-year-olds examined.

## Discussion

After the Second World War, there were no systematic data on the prevalence of caries in Europe. The data that first emerged primarily applied to the situation in western European countries (WEC) and Scandinavia, but little was known about caries prevalence in the countries of central and eastern Europe (CCEE).

The healthcare system in CCEE was set up so that it would be available to all residents to an equal degree; meanwhile, funding was insufficient for the best-quality and most expensive services. Social medicine thus had both positive and negative aspects. The national institutes of health – also in former Yugoslavia – largely did not maintain continuous records on the epidemiology of oral diseases. Therefore, many countries from the former Eastern Block did not have suitable data on the prevalence of caries. We started to gather data on caries prevalence in Yugoslavia in 1986 and continued the surveys in Slovenia up to the present.

As documented by the present report, a substantial decrease in the mean DMFT, as well as a significant increase in the number of sealed teeth per person was already achieved between the first two surveys. The effective caries preventive programme was granted the IAPD’s First Place Award and Certificate of Recognition for good oral health in children in Slovenia in 1997.^28^ The Slovenian Association of Paediatric Dentistry, the Slovenian Dental Association, and the Slovenian Oral Health Society contributed the most to the prevention of caries in Slovenia through their activities, as well as by promoting oral health and better awareness of self-care. The reduction in caries experience was largely ascribed to the widespread paediatric dentistry in every community, whereby all dentists, dental assistants, and preventive nurses implement a uniform professional concept. Dental services also include special dental clinics for children with special needs and pregnant women. The National Oral Health Education Programme (NOHEP) is part of the National Caries Preventive Programme. Thanks to the good organisation and distribution of PYHCs throughout the country, it was encouraging to observe that there are no significant differences between the urban and rural population for the first (p > 0.05) and last survey (p > 0.05) regarding caries experience (Table 5).

**Table 5:** Mean caries experience (DMFT), percentage of persons with sealed teeth and percentage of caries-free 12-year-olds in Slovenia by urbanisation in 1987 and 2017

	1987		2017	
	Urban	Rural	Urban	Rural
DMFT (SD)	4.9 (3.2)	5.3 (3.5)	1.4 (1.6)	1.7 (1.9)
Persons with sealed teeth (%)	10.5	1.5	96.7	90.7
Caries-free persons (%)	5.5	7.8	43.3	40.7

**Table 6:** Some factors determining the state of oral health at the national level at the beginning and end of the study^13,14,21,23,33^

Year	Total health expenditure as % of GDP	Total oral health expenditure as % of total health expenditure	Number of dentists per 100,000 inhabitants	Dentists who practice paediatric and adolescent dentistry as % of total number of dentists
1987	4.93	9.22	55	35
2017	8.19	4.74	70	27

Based on our findings, large-scale fissure sealing proved to be the most effective preventive measure in Slovenia, enabling us to reach almost 94% of 12-year-olds in the last survey, which is among the highest in the world.^16^ Thus, the preventive measures used in Slovenia contributed significantly to the decrease in the DMFT throughout the years.

It was possible to reach this high percentage because this preventive measure is implemented at all dental clinics located in primary schools and in Public Youth Health Centres (PYHC).

The second most widely used preventive measure in schoolchildren in Slovenia is a national competition with the slogan ‘Let’s have clean teeth,’ in which 99.8% of primary schools and 100% of special-needs schools participate. The competition started 35 years ago at one primary school in Ljubljana and has since spread throughout the country. This national competition is held by the Slovenian Dental Association, which received the FDI Smile Award in the Innovation category in 2017.^5^ Dentists practicing paediatric and adolescent dentistry treat children in all health regions of Slovenia.

Children with certain pathologies or patient issues that demand special knowledge and experience are referred to the paediatric dentistry specialists at the Centre for Paediatric and Preventive Dentistry at the Ljubljana University Medical Centre, which has state-of-the-art equipment and provides the most specialised oral health treatment for children. The paediatric dentistry specialists at this centre are actively involved in several international associations, which have established uniform professional guidelines for providing the best oral health treatment for children and adolescents.^7^

### Caries Prevalence of 12-year-olds in all Republics and Provinces of Former Yugoslavia since 1986

This section describes the caries situation among 12-year-olds in Slovenia in relation to the status of caries in the republics of the former Yugoslavia, now independent countries (Slovenia, Croatia, Bosnia and Herzegovina, Serbia, Montenegro and North Macedonia) and provinces: the Autonomous Province of Vojvodina and Kosovo, which is a partially recognised independent country. Given that the situation of Kosovo has not yet been resolved at the international level, we take a neutral position regarding this matter and in the following text we use the term ‘Kosovo’ to refer to the former Yugoslav province.

In 1986, a study was conducted by the representatives of all university faculties of stomatology in Yugoslavia in the form of the interrepublic research project ‘Oral health status and treatment needs of the Yugoslav population using basic criteria of the World Health Organization.’ Oral health and treatment need assessment forms (with CPITN) from 1982 combined by the WHO were used.^1^ Prior to the project, all the examiners were calibrated at a WHO seminar held in Bled, Slovenia, in 1985. The surveys were conducted in 22 Yugoslav towns, including 11 developed areas and 11 less developed areas. A total of 2600 persons in the age groups 6, 12, 15, 18, 35–44, and 65+ were examined.^26,31^

The first National Oral Pathfinder Survey (NOPS) was carried out in all republics and provinces of former Yugoslavia at the same time (in 3 months) in 1986,^31^ and it was decided to repeat the survey every 5 years. However, this happened only in Slovenia. In the other republics and provinces, no additional planned and organised surveys were undertaken because of the war and other difficult political and social circumstances.

Now, more than 30 years later, we have analysed the status of dental caries among 12-year-olds in the republics and provinces of former Yugoslavia by making contact with experts in those countries (mostly university professors who are paediatric dentistry specialists), who carried out epidemiological studies to various extents. The results of these studies are compiled and presented in Table 7. We compared all of the results obtained on DMFT from 2004 to 2017 with DMFT data for 12-year-olds from 1986 (NOPS), which were processed and published by the WHO Regional Office for Europe (Geneva).^31^ The overall caries experience in former Yugoslavia in 1986 was 6.1 DMFT, whereas the latest caries data available show a decline to 3.6 DMFT (Table 7).

**Table 7:** Comparison of the DMFT in 12-year-olds in all republics and provinces of former Yugoslavia from 1986 with the latest data obtained (2004–2017)

Basic DMFT data (1986)				Latest DMFT data (2004-2017)			
Republic/ province	Year	DMFT	Sample remarks	Year	n	DMFT	Sample remarks
Bosnia and Herzegovina	1986	6.2	NOPS	2004	560	4.2	N ➀
Croatia	1986	7.6	NOPS	2015	300	4.7	R ➁
Kosovo	1986	5.9	NOPS	2005	343	5.8	R ➂
Macedonia	1986	6.5	NOPS	2015	396	3.5	R ➃
Montenegro	1986	6.9	NOPS	2006	455	3.4	R ➄
Serbia	1986	5.0	NOPS	2008	225	2.6	R ➅
Slovenia	1986	6.1	NOPS	2017	300	1.5	NOPS
Vojvodina	1986	5.9	NOPS	2008	75	3.0	R ➅
Former Yugoslavia	1986	6.1	NOPS	2004-17	3,454	3.6	

N: national survey; R: random sample in the specified region; NOPS: National Oral Pathfinder Survey.* Circled numbers refer to reports about dental caries data for former Yugoslav republics and provinces; see Supplementary Material below.

A comparison of the mean DMFT between individual republics and provinces shows that in 1986, this average varied considerably between the highest mean DMFT 7.6 in Croatia and lowest value of 5.0 in Serbia. However, the latest data (2004–2017) show an even greater difference in means, from highest value of 5.8 in Kosovo to 1.5 in Slovenia. Slovenia managed to achieve the greatest reduction in the DMFT index, whereas Kosovo recorded a minimum decline of only 0.1, thus ranking last among former Yugoslav republics and provinces. The majority of former republics (Serbia, Montenegro, and North Macedonia) and the remaining province, Vojvodina, managed to reach almost half the mean DMFT, whereas Croatia and Bosnia and Herzegovina were unable to attain that kind of reduction. In summary, the DMFT index from 2004 to 2017 in individual republics and provinces shows an improvement over the last decades, but not to a satisfactory extent in all of them. Data on caries in 1986 were obtained simultaneously, using the same methodology in all of the republics and provinces of Yugoslavia; however, the study designs varied across nations between 2004 and 2017. This makes direct comparisons somewhat difficult. Nevertheless, it allows insight into more recent caries experience in the territory of the former common State (see Supplementary Material, below).

### Caries Prevalence of 12-year-olds in Slovenia in Comparison with Selected European Countries in the Last Decades

**Fig 4:**
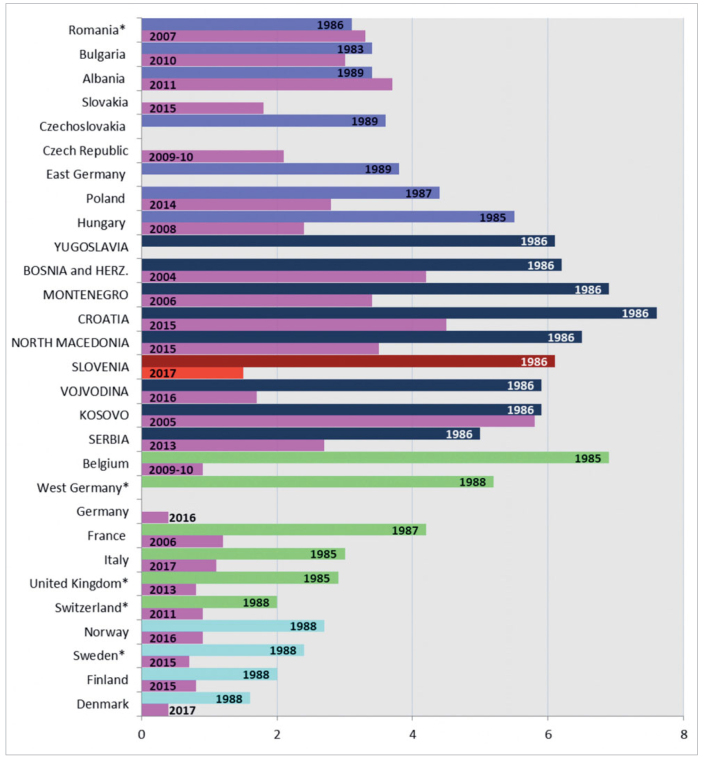
Comparison of the decrease in the DMFT index of 12-year-olds between Slovenia, former Yugoslavia, and selected European countries in the last 30 years. *Romania (2007): Constanta District (Urban Area); *Sweden (1988): DFT instead of DMFT; however, MT was certain to be smaller than 0.05; *Switzerland (2011): Canton of Basel-Landschaft; *United Kingdom (1985): caries data for England and Wales; *United Kingdom (2013): caries data for England, Wales and Northern Ireland.

In 1990, the 37th ORCA Congress took place in Ljubljana, Slovenia. At the symposium, the caries status of different age groups in 31 European countries was discussed. Later that year Thomas Marthaler gathered the findings on caries prevalence and published a symposium report, ‘Caries Status in Europe and Predictions of Future Trends’,^10^ which was considered the most comprehensive overview of caries prevalence in Europe after the Second World War at that time. This report serves as a data source for initial caries data, presenting the changes in DMFT index among 12-year-olds in Slovenia over the past 30 years in comparison with some other European countries during this time (Fig 4). The other initial caries data source is the WHO report mentioned above for Yugoslavia.^31^ The latest caries data for the former Yugoslav republics and provinces are used to compare Slovenia with other countries of the region, and were obtained from the authors listed in the Supplementary Material (below). The latest caries data for some other European countries are shown to compare the changes over time that occurred in caries prevalence among 12-year-olds between Slovenia and its broader European context. These caries data were mostly obtained from country oral health database of the WHO Global Oral Health Data Bank,^30^ except for Romania^15^ and Slovakia.^4^

In Fig 4, the first group of countries shows the caries levels in Central and Eastern Europe (CCEEs). From 1983 to 1989, the DMFT index was very high in Yugoslavia (DMFT 6.1), high in Hungary (DMFT 5.5), and moderate in Romania, Bulgaria, Czechoslovakia, East Germany, and Poland (DMFT 3.1–4.4) according to the WHO categories. In 1986, Slovenia had DMFT = 6.1 and in this group only four of the former Yugoslav republics had higher DMFT values. In the 1980s, the situation was quite different from that of Western European countries (WECs), where the DMFT index was mostly moderate (2–4.2), except for the high or very high values in West Germany (DMFT 5.2) and Belgium (DMFT 6.9). The only higher DMFT index was found for Croatia (DMFT 7.6), at that time still a Yugoslav republic. The third group of countries included Scandinavia, which demonstrated low DMFT values (DMFT 1.6–2.7). A few years later, on the basis of the surveys between 1991 and 1993, DMFT values for seven central European countries were reported to be lower; Austria, Bulgaria, the Czech Republic, Hungary, Poland, Romania, and Slovakia had moderate disease levels (DMFT 2.7–4.4), except for Poland with a high score (DMFT 5.1).^9^

In an international report from 2003,^18^ the mean DMFT values of 12-year-olds of six WECs and eight CCEEs (1996–2001) were compared based on data from oral health surveys carried out in these countries. The study revealed that the mean caries experience was higher in most CCEEs (DMFT around 3–4), except for Slovenia with a mean DMFT value <2. Meanwhile, the DMFT values declined significantly in WECs, although the disease level continued to be high in former East Germany compared to former West Germany. All of the WECs had a mean DT value <1, whereas most of the CCEEs had a mean DT around two. Of the WECs, Denmark and former West Germany, and of the CCEEs, Slovenia were the only three countries that had a mean DT < 0.5.

Finally, some Eastern European countries – with different economic and political systems – joined the EU in 2004. An analysis pointed out that the DMFT of 12-year-olds in those countries was higher in comparison to Western European countries, but only Slovenia and former East Germany achieved a substantial and continued decline in caries prevalence.^11^

The latest caries data (Fig 4) show that the only two countries of CCEE in which the DMFT index was higher post-2007 than in the late 1980s are Romania and Albania. Kosovo and Bulgaria recorded the smallest decrease in the DMFT index, while the largest decrease in the DMFT index by far was recorded in Belgium: the DMFT dropped from 6.9 to 0.9. Switzerland has experienced a caries reduction over the past 30 years similar to that observed in Scandinavia. In the late 1980s, Denmark had the lowest DMFT index, and its latest DMFT index is 0.4. This is the lowest figure; among European countries compared, only Germany was able to achieve this as well.

Although caries prevalence has decreased throughout Europe in the last 30 years, a reduction in caries did not occur in all European countries, nor did it reach the same extent among the countries. This is mainly because CCEEs had much higher caries prevalence to begin with, in addition to political and economic changes. Eastern European countries went through a transition, which made it even more difficult for them to keep up with the West in various aspects of improving the healthcare systems, including caries reduction.

## Conclusion

The present report documents the progress made in Slovenia in development of dental disease prevention within the context of the national public health scheme. Systematic disease prevention was incorporated into public dental health service, including outreach to all schoolchildren, the appropriate use of fluoride, pit and fissure sealing of permanent teeth, and raising the awareness of parents concerning their children’s dental health. The preventive efforts carried out by public health dentists, particularly the application of pit and fissure sealing, resulted in growing reductions of caries experience in 12-year-old children. The improvement in dental health of 12-year-olds in Slovenia was compared with the achievements over 30 years in key European countries with systematic dental health programmes for schoolchildren. The evidence of a caries prevalence decline in children of most European countries was reviewed. From an initial very high level of caries in schoolchildren, it is documented that the Slovenian preventive programme contributed to better dental health of children, similar to the level of European countries with advanced preventive dentistry and public health approaches. It is necessary to emphasise the need to preserve the national preventive programme. The introduction of modern systems for surveillance of dental health would be of paramount importance.
